# Risk factors for cervical lymph node metastasis of papillary thyroid cancer in elderly patients aged 65 and older

**DOI:** 10.3389/fendo.2024.1418767

**Published:** 2024-06-24

**Authors:** Yu Zhang, Xiaoyu Ji, Zhou Yang, Yu Wang

**Affiliations:** ^1^ Department of Head and Neck Surgery, Fudan University Shanghai Cancer Center, Shanghai, China; ^2^ Department of Oncology, Shanghai Medical College, Fudan University, Shanghai, China; ^3^ Department of Oncology, Huashan Hospital Fudan University, Shanghai, China

**Keywords:** papillary thyroid cancer, elderly, central lymph node metastasis, lateral lymph node metastasis, aging

## Abstract

**Objective:**

To assess the risk factors of cervical lymph node metastasis in elderly patients aged 65 years and older diagnosed with papillary thyroid cancer (PTC).

**Design and method:**

In this retrospective analysis, we included a total of 328 elderly patients aged 65 years and older diagnosed with PTC. We thoroughly examined clinical features from these patients. Utilizing univariate and multivariate logistic regression analyses, we aimed to identify factors contributing to the risk of central and lateral lymph node metastasis (CLNM/LLNM) in this specific population of PTC patients aged 65 years and older.

**Results:**

In the univariate analysis, CLNM was significantly associated with tumor size, multifocality, bilaterality, and microcalcification, while only tumor size ≥ 1cm (OR = 0.530, P = 0.019, 95% CI = 0.311 – 0.900) and multifocality (OR = 0.291, P < 0.001, 95% CI = 0.148 - 0.574) remained as risk factors in the multivariate analysis. LLNM was confirmed to be associated with male (OR = 0.454, P < 0.020, 95% CI = 0.233 - 0.884), tumor size ≥ 1cm (OR = 0.471, P = 0.030, 95% CI = 0.239 – 0.928), age ≥ 70 (OR = 0.489, P = 0.032, 95% CI = 0.254 – 0.941), and microcalcification (OR = 0.384, P = 0.008, 95% CI = 0.189 – 0.781) in the multivariate analysis. In elderly PTC patients with CLNM, male gender (OR = 0.350, P = 0.021, 95% CI = 0.143 – 0.855), age ≥ 70 (OR = 0.339, P = 0.015, 95% CI = 0.142 – 0.810), and bilaterality (OR = 0.320, P = 0.012, 95% CI = 0.131 – 0.779) were closely associated with concomitant LLNM in both univariate and multivariate analyses.

**Conclusion:**

For elderly PTC patients aged 65 and older, tumor size ≥ 1cm and multifocality are significant risk factors for CLNM. Meanwhile, male, tumor size ≥ 1cm, age ≥ 70, and microcalcification are crucial predictors for LLNM. In patients already diagnosed with CLNM, male, age ≥ 70, and bilaterality increase the risk of LLNM.

## Introduction

Papillary thyroid cancer (PTC) is the most common type of thyroid cancer, and its incidence has been increasing worldwide. While PTC is generally associated with a favorable prognosis and low malignancy, up to 50% of PTC patients present with clinically evident regional lymph node metastasis (LNM) at diagnosis (1, 2). The high incidence of lymph node metastasis in PTC is widely regarded as a significant factor contributing to cancer recurrence and poor prognosis (3, 4). The prevailing view currently is that for PTC patients, lymph node dissection should only be performed in cases where there is clear evidence of lymph node metastasis, such as confirmed by biopsy (5). Therefore, it is particularly important to thoroughly assess the risk of lymph node metastasis in patients preoperatively and guide treatment accordingly.

Current research suggests that lymph node metastasis in PTC patients is associated with several potential risk factors, including male gender, larger tumor size, multifocal tumors, extrathyroidal extension, and other factors (6–8). Central lymph node metastasis (CLNM) is highly prevalent among PTC patients. Surgical intervention of central lymph node dissection (CLND) is considered appropriate based on the recommendation of the American Thyroid Association, which advises cervical node dissection for patients presenting with clinically involved (cN1) cervical lymph nodes (5). Lateral lymph node metastasis (LLNM) usually follows the occurrence of CLNM, a sequence facilitated by the lymphatic drainage system. In certain cases of PTC, metastasis to the lateral neck lymph nodes (LLNs) can occur without involvement of the central lymph nodes. This may be attributed to the thyroid papillary carcinoma metastasizing to the LLNs via the superior thyroid artery, rather than through the classical lymphatic drainage system (9, 10).

It is widely recognized that age is a crucial factor influencing the prognosis of thyroid cancer patients. The 8th edition of the American Joint Committee on Cancer guidelines indicates that elderly patients have a lower disease-free survival rate (11). The aging population is increasing in many countries globally, elderly individuals with papillary thyroid cancer are not uncommon in clinical practice. Peri et al. proposed that elderly and young patients with thyroid cancer may have different pathogenic mechanisms (12). Studies have also indicated that the malignancy degree of thyroid nodules in individuals over 65 years old is closely associated with age (13, 14). The study by Zhou et al. demonstrated that active surgical treatment is beneficial for PTC patients under the age of 85 (15). Further investigation into elderly patients with papillary thyroid cancer, particularly the characteristics of neck lymph node metastasis in this subgroup, holds significant clinical significance.

This study analyzed the clinicopathological data of patients aged 65 and older with PTC, assessing risk factors associated with lymph node metastasis. The findings of this study can assist surgeons in evaluating cervical lymph nodes and making appropriate clinical decisions in elderly patients with papillary thyroid cancer preoperatively.

## Methods

The medical records of 519 patients aged 65 years and older who underwent partial or total thyroidectomy at Fudan University Shanghai Cancer Center (FUSCC) from January 2019 to December 2020 were reviewed. The study was approved by the Ethics Committee of FUSCC (050432–4-2307E), and informed consent was obtained from all patients.

Diagnostic evaluations and surgical management were conducted in accordance with the guidelines of the American Thyroid Association (ATA). Patients were included based on the following criteria: 1) Confirmation of PTC through surgical pathology, 2) Pathological confirmation of the presence or absence of LNM, 3) Availability of complete clinical data, and 4) Age ≥65 years for all 519 patients. A total of 191 patients were excluded based on the following criteria: 1) Benign final pathological findings, 2) Pathological diagnosis of thyroid malignancies other than PTC, 3) Incomplete clinical data, 4) History of prior thyroid surgery, 5) Presence of other extrathyroidal malignancies, and 6) History of head and neck radiation therapy. Ultimately, 328 patients were included in this study. All participants included in the study underwent ultrasound-guided fine-needle aspiration biopsy (UG-FNAB) several weeks prior to surgery. Tumor morphology-related ultrasound data, including microcalcifications, were obtained by ultrasonography examination performed by two experienced radiologists specializing in thyroid pathology. Following surgery, diagnosis confirmation and corresponding histopathological features were assessed by two experienced thyroid tumor pathologists.

Clinical and pathological characteristics data were collected, including age, sex, tumor size, capsular invasion, tumor location, unifocality/multifocality, unilateral/bilateral involvement, presence of Hashimoto’s thyroiditis, presence of nodular goiter, tumor margin smoothness, presence of microcalcifications, central compartment lymph node metastasis, and lateral compartment lymph node metastasis.

Data management and statistical analyses were conducted using IBM SPSS Statistics for Windows, Version 21.0 (IBM Corp.). Continuous variables such as age and tumor size were summarized using means and variances, with group comparisons conducted through chi-square or non-parametric U tests. Categorical variables were described using frequency percentages, and group comparisons were made using the Chi-square test. Initial analyses involved univariate assessment using either the chi-squared test or Fisher’s exact test. Variables with a significance level of p < 0.05 underwent subsequent multivariate analyses utilizing binary logistic regression. The data evaluating the influence of potential risk factors are reported as odds ratios (ORs) with corresponding 95% confidence intervals (CIs).

## Results

### Patient characteristics

In our study, 328 patients aged 65 years and older diagnosed with papillary thyroid cancer (PTC) were included. The mean age (± standard deviation) was 68.4 ± 3.2 years (range, 65–80 years), with 75.3% of the patients being female (247 females and 81 males). Among all 328 elderly PTC patients, central neck lymph node metastasis (CLNM) occurred in 109 patients, lateral neck lymph node metastasis (LLNM) occurred in 60 patients, and among the 109 patients with CLNM, 46 patients developed LLNM. **Tables 1** **,** **2** show the clinical and ultrasonic factors of the patients.

**Table 1:** Association between CLNM and clinical characteristics of elder PTC patients.

Variables	CLNM(%)		P value
	- (N=219)	+ (N=109)	
Gender			0.098
Female	171 (69.28)	76 (30.72)	
Male	48 (59.26)	33 (40.74)	
Tumor size (cm) <1 ≥1 Age 65≤age<70 age≥70	117 (75.99) 102 (58.62) 162 (68.92) 57 (61.95)	37 (24.01) 72 (41.38) 73 (31.08) 36 (38.05)	0.001 0.185
Location			0.431
Upper	61 (64.91)	33 (35.09)	
Middle Lower Full	82 (66.67) 69 (71.15) 7 (50.00)	41 (33.33) 28 (28.85) 7 (50.00)	
Multifocality			0.001
Yes	58 (50.43)	57 (49.57)	
No	161 (75.59)	52 (24.41)	
Coexistent HT Yes	56 (75.68)	18 (24.32)	0.065
No	163 (64.14)	91 (35.86)	
Coexistent NG Yes	102 (69.84)	44 (30.16)	0.287
No	117 (64.28)	65 (35.71)	
ETE			0.121
Yes No Bilateral Yes No	23 (56.10) 196 (68.30) 35 (51.52) 184 (70.78)	18 (43.90) 91 (31.70) 33 (48.48) 76 (29.22)	0.003
T T1–2	180 (69.23)	80 (30.77)	0.064
T3–4	39 (57.35)	29 (42.65)	
Margin			0.492
Smooth	60 (69.77)	26 (30.23)	
Ill-defined	159 (65.69)	83 (34.31)	
Microcalcification Yes	114 (60.67)	74 (39.33)	0.006
No	105 (75.00)	35 (25.00)	

CLNM, central lymph node metastasis; PTC, papillary thyroid cancer; HT, Hashimoto’s thyroiditis; NG, Nodular Goiter; ETE, Extrathyroidal Extension.

**Table 2:** Association between LLNM and clinical characteristics of elder PTC patients with central lymph node metastasis.

Variables	LLNM in CLNM(%)		P value
	- (N=63)	+ (N=46)	
Gender			0.032
Female	49 (64.53)	27 (35.47)	
Male	14 (42.42)	19 (57.58)	
Tumor size (cm) <1 ≥1 Age 65≤age<70 age≥70	24 (64.86) 39 (54.17) 48 (65.75) 15 (41.67)	13 (35.14) 33 (45.83) 25 (34.25) 21 (58.33)	0.284 0.017
Location			0.134
Upper	16 (48.48)	17 (51.52)	
Middle Lower Full	28 (68.29) 17 (60.71) 2 (28.57)	13 (31.71) 11 (39.29) 5 (71.43)	
Multifocality			0.126
Yes	29 (50.88)	28 (49.12)	
No	34 (65.38)	18 (34.62)	
Coexistent HT Yes	9 (50.00)	9 (50.00)	0.463
No	54 (59.34)	37 (40.66)	
Coexistent NG Yes	30 (68.18)	14 (31.82)	0.071
No	33 (50.78)	32 (49.22)	
ETE			0.833
Yes No Bilateral Yes No	10 (55.56) 53 (58.26) 13 (39.39) 50 (65.79)	8 (44.44) 38 (41.74) 20 (60.61) 26 (34.21)	0.010
T T1–2	46 (57.54)	34 (42.46)	0.917
T3–4	17 (58.62)	12 (41.38)	
Margin			0.356
Smooth	13 (50.00)	13 (50.00)	
Ill-defined	50 (60.24)	33 (39.76)	
Microcalcification Yes	39 (52.70)	35 (47.30)	0.117
No	24 (68.57)	11 (31.43)	

CLNM, central lymph node metastasis; LLNM, lateral lymph node metastasis; PTC, papillary thyroid cancer; HT, Hashimoto’s thyroiditis; NG, Nodular Goiter; ETE, Extrathyroidal Extension.

### Association of CLNM/LLNM and clinical characteristics of elder PTC patients aged 65 years and older

In the univariate analysis of CLNM with clinical/ultrasound features in all elderly PTC patients (see **Table 1**), CLNM was found to be significantly associated with tumor size (P < 0.001), multifocality (P < 0.001), bilaterality (P = 0.003), and ultrasound-detected microcalcifications (P = 0.006). However, no significant association was observed between CLNM and other clinical factors such as Gender (P = 0.098), HT (P = 0.065), etc.

In the univariate analysis of LLNM with clinical/ultrasound features in all elderly PTC patients (see **Table 3**), LLNM was significantly associated with Gender (P = 0.017), tumor size (P < 0.001), age (P = 0.011), tumor location (P = 0.003), multifocality (P < 0.001), bilaterality (P < 0.001), and microcalcifications (P < 0.001). However, no significant association was observed between LLNM and other clinical factors such as ETE (P = 0.131), HT (P = 0.600), NG (P = 0.054), etc.

**Table 3:** Association between LLNM and clinical characteristics of elder PTC patients.

Variables	LLNM (%)		P value
	- (N=268)	+ (N=60)	
Gender			0.017
Female	209 (84.60)	38 (15.40)	
Male	59 (72.83)	22 (27.17)	
Tumor size (cm) <1 ≥1 Age 65≤age<70 age≥70	138 (89.66) 130 (74.70) 200 (85.11) 68 (73.00)	16 (10.34) 44 (25.30) 35 (14.89) 25 (27.00)	0.001 0.011
Location			0.003
Upper	72 (76.60)	22 (23.40)	
Middle Lower Full	105 (85.42) 84 (86.57) 7 (50.00)	18 (14.58) 13 (13.43) 7 (50.00)	
Multifocality			0.001
Yes	81 (70.45)	34 (29.55)	
No	187 (87.80)	26 (12.20)	
Coexistent HT Yes	62 (83.78)	12 (16.22)	0.600
No	206 (81.14)	48 (18.86)	
Coexistent NG Yes	126 (86.84)	20 (13.16)	0.054
No	142 (78.02)	40 (21.98)	
ETE			0.131
Yes No Bilateral Yes No	30 (73.17) 238 (82.92) 42 (61.76) 226 (86.92)	11 (26.83) 49 (17.08) 26 (38.24) 34 (13.08)	0.001
T T1–2	217 (83.46)	43 (16.54)	0.108
T3–4	51 (75.00)	17 (25.00)	
Margin			0.574
Smooth	72 (83.72)	14 (16.28)	
Ill-defined	196 (81.00)	46 (19.00)	
Microcalcification Yes	142 (75.54)	46 (24.46)	0.001
No	126 (90.00)	14 (10.00)	

LLNM, lateral lymph node metastasis; PTC, papillary thyroid cancer; HT, Hashimoto’s thyroiditis; NG, Nodular Goiter; ETE, Extrathyroidal Extension.

For elderly PTC already with CLNM (see **Table 2**), the univariate analysis revealed significant associations between LLNM and Gender (P = 0.032), age (P = 0.017), and bilaterality (P = 0.010). Nevertheless, no significant relationship was found between LLNM and other clinical factors including multifocality (P = 0.126), microcalcifications (P = 0.117), NG (P = 0.071), among others.

### Multivariate logistic regression analysis

In the multivariate analysis, CLNM in elderly PTC patients was significantly associated with tumor size ≥ 1cm (OR = 0.530, P = 0.019, 95% CI = 0.311 – 0.900) and multifocality (OR = 0.291, P <0.001, 95% CI = 0.148 - 0.574), as shown in **Table 4**. Regarding LLNM, male gender (OR = 0.454, P <0.020, 95% CI = 0.233 - 0.884), tumor size ≥ 1cm (OR = 0.471, P = 0.030, 95% CI = 0.239 – 0.928), age ≥ 70 (OR = 0.489, P = 0.032, 95% CI = 0.254 – 0.941), and microcalcifications (OR = 0.384, P = 0.008, 95% CI = 0.189 – 0.781) were identified as closely associated risk factors in elderly PTC patients, as shown in **Table 5**. For elderly PTC patients with CLNM, male gender (OR = 0.350, P = 0.021, 95% CI = 0.143 – 0.855), age ≥ 70 (OR = 0.339, P = 0.015, 95% CI = 0.142 – 0.810) and bilaterality (OR = 0.320, P = 0.012, 95% CI = 0.131 – 0.779) were found to be closely associated risk factors for concomitant LLNM, as shown in **Table 6**.

**Table 4:** Multivariate logistic regression analyses of factors contributing to central lymph node metastasis in elder PTC patients.

Characteristics	OR	P Value	95% CI for OR	
			Lower	Upper
Size ≥ 1 Multifocality	0.530 0.291	0.019 <0.001	0.311 0.148	0.900 0.574

CI, confidence interval; OR, Odds ratio.

**Table 5:** Multivariate logistic regression analyses of factors contributing to lateral lymph node metastasis in elder PTC patients.

Characteristics	OR	P Value	95% CI for OR	
			Lower	Upper
Male Size ≥1 Age ≥70 Microcalcification	0.454 0.471 0.489 0.384	0.020 0.030 0.032 0.008	0.233 0.239 0.254 0.189	0.884 0.928 0.941 0.781

CI, confidence interval; OR, Odds ratio.

**Table 6:** Multivariate logistic regression analyses of factors contributing to lateral lymph node metastasis in elder PTC patients with central lymph node metastasis.

Characteristics	OR	P Value	95% CI for OR	
			Lower	Upper
Male Age ≥ 70 Bilateral	0.350 0.339 0.320	0.021 0.015 0.012	0.143 0.142 0.131	0.855 0.810 0.779

CI, confidence interval; OR, Odds ratio.

## Discussion

In this study focusing on elderly PTC patients aged 65 years and older, we demonstrated that tumor size larger than 1cm and multifocality were risk factors for CLNM. Additionally, male gender, Tumor size ≥ 1cm, age ≥ 70, and microcalcifications were closely associated with LLNM. For patients who already presented with CLNM, male gender, age ≥ 70, and bilaterality significantly increased the risk of further LLNM.

The current research holds that age is closely associated with the prognosis of PTC patients. In the previous edition of the AJCC system, forty-five serves as a significant age threshold for prognostic risk assessment (16). For patients with differentiated thyroid cancer under the age of 45, even with distant metastasis, they are still classified as stage II. However, recent studies have raised doubts about the use of age 45 as a cutoff to upstage patients (17, 18). One significant factor influencing the designation of 45 years as a prognostic risk threshold for thyroid cancer patients may be the significant impact of lymph node metastasis on prognosis (19). Therefore, for PTC patients, age and lymph node metastasis are risk factors that need to be considered comprehensively. Recently, in the eighth edition of the AJCC TNM classification system, the age cutoff for PTC was adjusted from 45 to 55 years, following analysis of several population-based studies (20, 21). According to this revision, the age cutoff of 55 years is more robust, leading to better predictability for cancer-specific survival (22, 23).

The World Health Organization (WHO) typically defines individuals aged 65 and above as elderly. With the increasing size of the aging population, the potential PTC patients aged 65 and above cannot be ignored. Prioritizing the risk of lymph node metastasis in elderly PTC patients is particularly important for prognosis management in this demographic group. In this study, we found that for CLNM, age in elderly patients is not a significant risk factor. However, for LLNM, including cases where CLNM already exists, older age is a key factor in promoting LLNM occurrence. This aligns with current research perspectives, as noted by Wang et al., who indicated that the protective effect of age on cervical LNM diminishes gradually and increasingly with age, particularly for lateral LNM (24). Elderly patients frequently exhibit poorer health conditions compared to younger patients, often accompanied by multiple underlying diseases. Once cervical lymph node metastasis occurs, elder patients face increased treatment-related risks and poorer prognosis, hence requiring particular attention.

According to previous studies, male gender, larger tumor size, multifocality, and extrathyroidal extension are the main risk factors for lymph node metastasis in PTC (25–28). This study yielded similar results. In elderly thyroid cancer patients, larger tumor size and multifocality are closely associated with an increased risk of CLNM occurrence. Tumor size is a critical component in TNM staging, with lesions larger than 1 cm demonstrating increased invasiveness as they grow in size (29). In some elderly patients, irregular medical examinations may lead to a longer potential growth period of the tumor, resulting in a larger tumor size at diagnosis. As for multifocality, reported rates of its occurrence in PTC range from 20.0% to 36.1% (30). In our study, multifocality was observed in 35.1% of elderly patients. This relatively high incidence underscores the significance of not missing potential multifocal lesions during preoperative assessment.

In elderly male PTC patients, the risk of LLNM is significantly higher. Male patients often exhibit advanced stage disease and aggressive histopathology (31). Furthermore, male patients with PTC tend to have worse prognoses than females diagnosed under the age of 55 years (32). In our study, LLNM incidence among elderly male PTC patients was 27.2%, nearly twice that of female patients. This underscores the importance of paying special attention to the potential risk of LLNM in elderly male PTC patients. Additionally, preoperative ultrasound findings indicating tumor microcalcification should also be given attention for potential LLNM risk. Microcalcification is a highly specific indicator and significant sonographic feature suggestive of malignant nodules and may increase the risk of cervical lymph node metastasis (33). In this study, elderly PTC patients with microcalcifications had a LLNM incidence of 24.5%, significantly higher than those without this risk feature. For elderly PTC patients with existing CLNM, bilateral lesions are a significant LLNM risk factor that should not be overlooked, consistent with findings from multiple studies (34, 35). In this study, 14 elderly PTC patients developed LLNM in the absence of CLNM, which accounts for nearly 25% of LLNM cases, aligning with previous research findings ranging from 5% to 25% (36, 37), this underscores the importance of preoperative assessment and warrants further investigation into the necessity of central compartment dissection in this subgroup.

In this study, capsule invasion did not correlate with the risk of lymph node metastasis. One possible explanation for this phenomenon is that lesions remained stable over time, even in the presence of capsule invasion, which may significantly increase the risk of tumor progression. Additionally, in our study, we found that patients with coexisting Hashimoto’s thyroiditis had a lower probability of lymph node metastasis, although not statistically significant, which is consistent with the current research findings (7, 38). There are differences in the occurrence of LLNM based on tumor location, particularly with tumors located in the upper pole of the thyroid gland showing a higher rate of lateral cervical lymph node metastasis. This finding aligns with the conclusions drawn from the present study (9, 39).

Our study is subject to certain limitations. Firstly, its retrospective design restricted the adjustment for certain confounding factors. Secondly, being a single-center analysis, a multicenter approach would be required for a more comprehensive understanding of this issue. At the same time, we did not incorporate molecular pathological features such as BRAF mutations, mainly due to the significant number of patients referred from external institutions already diagnosed through fine-needle aspiration, where BRAF testing was not universally conducted. Currently, we have placed considerable emphasis on collecting molecular pathological data for admitted patients. Additionally, with the increasing importance placed on molecular pathology, most medical institutions in the region now include testing for gene mutations such as BRAF when diagnosing PTC through fine-needle aspiration. In further studies, we will thoroughly incorporate molecular pathological information including BRAF mutations.

## Conclusion

This study, based on PTC patients aged 65 and older treated at our center, revealed several significant risk factors for CLNM, including tumor size ≥ 1cm and multifocality. Meanwhile, for LLNM, male gender, tumor size ≥ 1cm, age ≥ 70, and microcalcification on ultrasound are important risk factors. In patients who already have CLNM, male gender, age ≥ 70, and bilaterality are risk factors for LLNM.

## Data availability statement

The raw data supporting the conclusions of this article will be made available by the authors, without undue reservation.

## Ethics statement

The studies involving humans were approved by the Ethics Committee of Fudan University Shanghai Cancer Center (050432-4-2307E). The studies were conducted in accordance with the local legislation and institutional requirements. The participants provided their written informed consent to participate in this study.

## Author contributions

YZ: Data curation, Formal analysis, Writing – original draft. XJ: Conceptualization, Investigation, Methodology, Software, Writing – review & editing. ZY: Funding acquisition, Investigation, Project administration, Visualization, Writing – review & editing. YW: Funding acquisition, Project administration, Supervision, Validation, Writing – review & editing.
